# Metabolic-Associated Fatty Liver Disease, Hepatitis B Surface Antigen Seroclearance, and Long-Term Risk of Hepatocellular Carcinoma in Chronic Hepatitis B

**DOI:** 10.3390/cancers14236012

**Published:** 2022-12-06

**Authors:** Ming-Whei Yu, Chih-Lin Lin, Chun-Jen Liu, Wan-Jung Wu, Jui-Ting Hu, Yi-Wen Huang

**Affiliations:** 1 Graduate Institute of Epidemiology and Preventive Medicine, College of Public Health, National Taiwan University, Taipei 10055, Taiwan; 2 Department of Gastroenterology, Ren-Ai Branch, Taipei City Hospital, Taipei 10629, Taiwan; 3 Division of Gastroenterology, Department of Internal Medicine, National Taiwan University Hospital and Graduate Institute of Clinical Medicine, National Taiwan University College of Medicine, Taipei 10002, Taiwan; 4 Liver Center, Cathay General Hospital Medical Center, School of Medicine, Fu-Jen Catholic University College of Medicine, Taipei 24352, Taiwan; 5 Clinical Research Center, Liver Center and Division of Gastroenterology, Department of Internal Medicine, Taipei Medical University Hospital, Taipei 11031, Taiwan; 6 Division of Gastroenterology, Department of Internal Medicine, School of Medicine, College of Medicine, Taipei Medical University, Taipei 11031, Taiwan

**Keywords:** all-cause mortality, diabetes, extrahepatic mortality, hepatic steatosis, MBOAT7, metabolic dysfunction, obesity, PNPLA3, TM6SF2

## Abstract

**Simple Summary:**

Concomitant hepatic steatosis is common in patients with chronic hepatitis B (CHB), but data regarding its impact on the natural history of CHB remain sparse and conflicting. Recently, metabolic-associated fatty liver disease (MAFLD) was defined as hepatic steatosis in the presence of metabolic abnormality. The utility of MAFLD and its ability to assess hepatocellular carcinoma (HCC) risk for CHB requires further investigation. In this population-based cohort study of CHB, MAFLD was not associated with an increased risk of HCC. However, it was associated with a higher probability of functional cure for CHB in terms of HBsAg loss, and the MAFLD–HCC association was largely mediated by the development of HBsAg seroclearance driven by hepatic steatosis. On the contrary, the burden of metabolic risk abnormality was robustly associated with an increased risk of HCC in CHB, and this association was observed for participants both with and without hepatic steatosis and for participants both with and without HBsAg seroclearance.

**Abstract:**

The value of metabolic-associated fatty liver disease (MAFLD) and its ability to assess hepatocellular carcinoma (HCC) risk remains uncertain for chronic hepatitis B (CHB). We evaluated the impacts of MAFLD and its coincidental metabolic abnormalities and related genetic predisposition on HCC incidence and mortality outcomes in CHB. We analyzed data from 1453 HBsAg-positive men (median age = 49.2 years at baseline) from a cohort of civil servants recruited from 1989–1992. MAFLD was defined as hepatic steatosis on ultrasound with obesity, diabetes, or metabolic dysfunction at baseline. During follow-up (median = 19.3 years), 105 HCC events occurred. MAFLD was not associated with HCC (adjusted hazard ratio (aHR) = 1.02) but was associated with a higher HBsAg seroclearance rate (aHR = 1.43). In mediation analysis, HBsAg seroclearance driven by hepatic steatosis explained 31.6% of the association between MAFLD and HCC. Antiviral treatment or fatty liver disease-associated genetic variants did not influence the MAFLD–HCC association. In contrast, even after adjustment for MAFLD and the other metabolic abnormalities, diabetes (aHR = 2.28), obesity (aHR = 1.72), and metabolic dysfunction (aHR = 3.30) increased the risk of HCC (all *p* < 0.030). The risk of HCC increased with the number of metabolic abnormalities (vs 0: aHR = 2.05 and 5.72 for 2 and ≥ 3 metabolic abnormalities, respectively), and the cumulative effect of metabolic abnormalities was found across subgroups categorized by hepatic steatosis as well as in participants both with and without HBsAg seroclearance. In conclusion, MAFLD was not associated with increased HCC incidence in CHB. A more informative assessment of HCC risk can be obtained by taking into account the number of metabolic abnormalities.

## 1. Introduction

The prevalence of non-alcoholic fatty liver disease (NAFLD) has been increasing rapidly globally in recent years. It may become a major cause of liver-related complications and deaths related to metabolic risks [1]. The close interrelationship between NAFLD and most features of metabolic syndrome has been well documented [1,2], highlighting the important roles of metabolic risk factors in determining NAFLD-related disease burden. Recently, a panel of international experts proposed a new nomenclature, metabolic-associated fatty liver disease (MAFLD), to reflect metabolic dysfunction-associated fatty liver disease as being a change from NAFLD [3]. The definition of NAFLD includes evidence of hepatic steatosis, an absence of secondary causes of hepatic steatosis (e.g., excess alcohol consumption), and no concomitant liver diseases, while the definition of MAFLD requires metabolic risk factors in the setting of hepatic steatosis and does not exclude other etiologies of chronic liver disease. Large prospective studies, with adjustments for coexistent metabolic risk factors in different magnitudes, have demonstrated that MAFLD is associated with increased all-cause mortality and/or cardiovascular mortality, and most of these studies have reported that the effect of MAFLD is greater compared to that of NAFLD [4,5,6,7].

So far, there is limited information about the long-term risks of incident hepatocellular carcinoma (HCC) and liver-related mortality in relation to MAFLD [8]. Considering that MAFLD may coexist with other, distinct liver diseases, ranging from alcohol-associated liver diseases to viral hepatitis, it is reasonable to assume that the presence of other concomitant etiologies is pivotal for predicting liver-related outcomes. Indeed, a recent study using NHANES data, a representative sample of the U.S. population, suggested that the increased liver-related mortality among MAFLD is primarily driven by alcohol-associated liver diseases [9]. Furthermore, the natural history of patients with MAFLD in whom another etiology is the predominant cause of liver disease may be very different from that of those with pure metabolic dysfunction. In particular, more than 240 million individuals worldwide are chronically infected with the hepatitis B virus (HBV) [10]. Concurrent NAFLD is common in patients with chronic hepatitis B (CHB) [11], underlining the need to understand the interplay between both diseases. While the aggregate effect of metabolic risk factors has been associated with an increased risk of HCC in CHB [12], the presence of hepatic steatosis in CHB was associated with a higher incidence of hepatitis B surface antigen (HBsAg) seroclearance and a decreased risk of HCC [13,14]. It may seem obvious that the management of CHB patients with concomitant MAFLD should differ from that of uninfected patients.

Although CHB accounts for >50% of global burden of HCC incidents [10], information about the clinical impact of MAFLD on its applicability to identify HBV carriers at risk for HCC is sparse [8]. Evaluation of the long-term outcomes of MAFLD is complex because it is influenced by the presence of metabolic risk abnormalities and fatty liver disease-associated genetic predisposition. Therefore, using a population-based study of CHB with substantial 19-year follow-up data, we aimed to understand the roles of MAFLD, coincidental metabolic abnormalities, and fatty liver disease-related genetic predisposition in the natural course of HCC and their predictive values for long-term outcomes in CHB.

## 2. Patients and Methods

### 2.1. The Cohort

The participants were from a previously established cohort study consisting of 2878 male, HBsAg-positive civil servants aged ≥ 30 years at baseline who were recruited from the Government Employees’ Central Clinics (GECC) during routine free physical examination between 1989 and 1992 [11,12]. The GECC provided a general medical examination, physiological and anthropometric measurements, a chest x-ray, an electrocardiogram, and urine routine and blood testing, including blood count, HBsAg, and alanine aminotransferase (ALT) testing at all age levels. Those aged 40 years or above were additionally examined with aspartate aminotransferase (AST) and cardiovascular and renal risk seromarkers. Participants were followed longitudinally through two methods: (1) directly, by means of medical examinations including ultrasonography and blood testing; (2) indirectly, through linkages to the National Cancer Registry, the National Death Certification, and the National Registry for Catastrophic Illness Patient Database. The general design and operation of the cohort study have been previously published [11,12,13]. The study was approved by the National Taiwan University ethics committee (NTU-REC No.: 201912HM015) and all participants provided informed consent.

**Figure 1:**
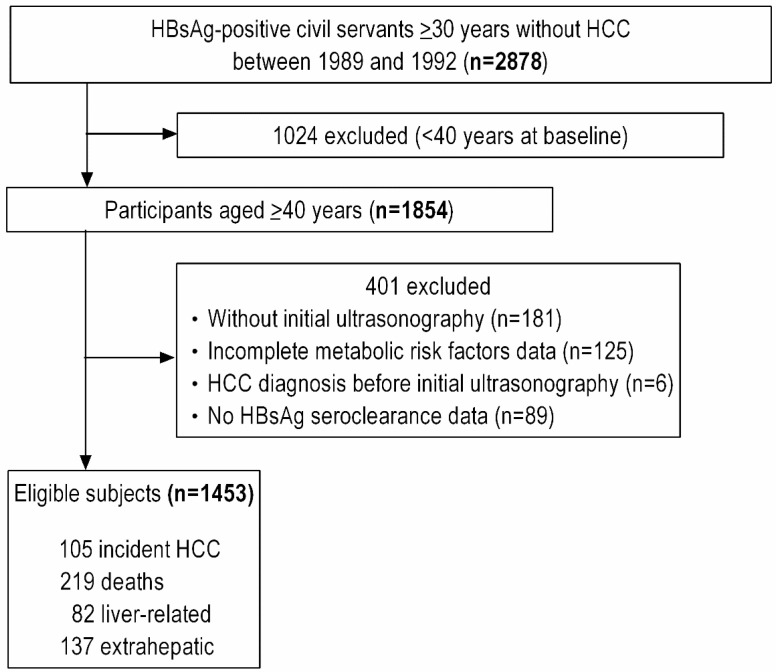
Flowchart of study population selection. HCC: hepatocellular carcinoma.

The present work is based on the analysis of the data from participants aged ≥ 40 years in whom metabolic risk factors were assessed at baseline. Among 1854 participants aged ≥ 40 years at enrollment, we excluded 181 individuals who did not undergo ultrasonography within the first three years after starting ultrasound follow-up and 125 individuals with missing data on one or more metabolic risk factors. We also excluded six individuals who had been diagnosed with HCC prior to the initial ultrasonography and those who had missing data on HBsAg seroclearance (n = 89), resulting in a final sample size of 1453 (Figure 1).

### 2.2. Clinical Evaluation

At baseline, the collected metabolic factors included body mass index (BMI), blood pressure, history of diabetes, and serum levels of glucose, triglycerides, and total cholesterol. Participants were interviewed according to a structured questionnaire including items of sociodemographic characteristics, lifestyle habits, and medical history. Serum HBsAg was tested using a radioimmunoassay (Abbott Laboratories, North Chicago, IL, USA), and anti-HCV was assayed using a second-generation enzyme immunoassay (Abbott Laboratories). Serum α-fetoprotein was measured using an enzyme-linked immunosorbent assay (Abbott Laboratories). To enable monitoring of liver-related events, participants were invited to return for follow-up clinical evaluation, including a repeated questionnaire interview, anthropometric assessment, abdominal ultrasonography, and blood biochemical tests. High-resolution, real-time ultrasonography has been performed routinely for follow-up examination since 1993.

In Taiwan, reimbursement under the National Health Insurance program for hepatitis B patients who meet antiviral therapy criteria began in 2003. The National Health Insurance Research Database (NHIRD), which consists of population-level data established for public research since 2002, includes information on outpatient visits and in-patient care, prescription drugs, and medical institutions [15]. We obtained information on anti-HBV therapy (interferons and nucleos(t)ide analogues) through a follow-up interview questionnaire and the linkage of our survey data with the NHIRD.

### 2.3. Definitions of Key Variables

Obesity (BMI ≥ 25 kg/m^2^) was defined by the criteria established for Asians [3]. Diabetes was defined in subjects with a history of diabetes diagnosis and/or treatment or a fasting blood glucose ≥ 126 mg/dL. Pre-diabetes was diagnosed when a fasting glucose level was between 100 and 125 mg/dL. Hypertriglyceridemia was defined as a triglycerides level ≥ 150 mg/dL. Hypercholesterolemia was defined as a total cholesterol level ≥ 240 mg/dL. High blood pressure was defined as a systolic/diastolic blood pressure ≥ 130/85 mm Hg.

Hepatic steatosis was detected via hepatic ultrasound. MAFLD was defined as the presence of hepatic steatosis and any one of the following three conditions: obesity, type 2 diabetes, or evidence of metabolic dysfunction. Metabolic dysfunction was defined as the presence of at least two metabolic risk abnormalities, including hypertriglyceridemia and high blood pressure.

### 2.4. HBsAg Seroclearance during Follow-Up

HBsAg loss is an important milestone in the natural history of CHB [16,17]. To determine the date of HBsAg seroclearance, serial follow-up plasma samples obtained since their original 1989–1992 entries into the study were retrieved to retrospectively assess for HBsAg; anti-HBs titers were further examined for subjects who became HBsAg seronegative, using Abbott Architect HBsAg and anti-HBs assays (Abbott Laboratories). The time of HBsAg seroclearance was defined as the first time of detection of seronegative HBsAg.

### 2.5. Laboratory Analyses

We selected three single nucleotide polymorphisms that are major genetic risk factors for NAFLD/fatty liver disease: PNPLA3 rs738409, TM6SF2 rs58542926, and MBOAT7 rs641738 [18]. Genotyping was performed using the TaqMan assay and QuantStudio 7 Flex Real-Time PCR System (Applied Biosystems, Foster City, CA, USA).

### 2.6. Outcomes

The incidence of HCC was the primary endpoint. All-cause and extrahepatic mortality were secondary endpoints.

### 2.7. Statistical Analysis

Descriptive statistics of included variables were examined in participants with and without MAFLD. We calculated the follow-up time from the date of recruitment to the date the outcome occurred, the date of death, or the end of the follow-up period, whichever came first. The analysis in this report was restricted to the cancer occurrence and mortality data from baseline examination (1989–1992) to 31 December 2010, with up to 21 years of follow-up [12]. The Cox proportional hazards model was used to estimate adjusted-hazard ratios (HRs) and 95% confidence intervals (CIs), as well as obtain adjusted cumulative incidence curves. Although metabolic abnormalities were included in the definition of MAFLD, each variable (such as diabetes, obesity, and metabolic dysfunction) may have its own effect on the risk of HCC. Therefore, the evaluation of the HCC risk in relation to MAFLD is complex and influenced by the heterogeneity in the metabolic risk factor profile between individuals. To address whether MAFLD per se was associated with HCC risk, three models were developed: (1) Model 1 adjusted for age; (2) Model 2, further adjusted for hepatic steatosis at initial ultrasonography and time-varying HBsAg seroclearance, which was treated as a time-dependent covariate; and (3) Model 3, further adjusted for tobacco and alcohol use, fatty liver disease-related genetic variants, and metabolic risk factors. Because of the biological relationship between MAFLD, hepatic steatosis, obesity, diabetes, and metabolic dysfunction, these variables were intercorrelated; two of the ten pairs had Pearson’s correlation coefficients >0.5 (MAFLD vs. obesity 0.70; MAFLD vs. hepatic steatosis 0.51) and the remaining pairs had Pearson’s correlation coefficients <0.23. However, the corresponding variance inflation factors after regressing each variable on all the other variables, which ranged from 1.0 to 3.1, were below the suggested threshold (≤5), indicating no notable multicollinearity that needed to be corrected.

We performed model-based mediation analysis using the method originally developed by Imai et al. in the R mediation package [19] to estimate the extent to which MAFLD may influence HBsAg seroclearance driven by hepatic steatosis and, in turn, affect the risk of developing HCC. In this analysis, the mediation proportion estimated indicates how much of the effect of MAFLD can be explained by the indirect path through hepatic steatosis and HBsAg seroclearance on HCC risk. All statistical analyses were conducted in SAS version 9.4 or R version 4.2.1. All *p*-values were obtained from two-tailed tests.

### 2.8. Sensitivity Analysis

We used a case-cohort study nested in the cohort of this study to examine the impacts of HBV genotype and viral load on the estimated effect of MAFLD on the risk of HCC. Data were from a previous case-cohort study involving 1143 CHB patients in the GECC cohort; for each subject, all major viral factors were measured at baseline [20]. We included all of the 593 CHB patients who were also included in the present work of MAFLD and HCC (Supplementary Materials).

To study the influence of the initial age of subjects and the missing information on waist circumference on the association between MAFLD and HCC, we also conducted sensitivity analyses using the longitudinal cohort data after waist circumference as part of the follow-up examination since 2006. In the analysis, we referred to the first waist circumference assessment during the observational period (2006–2021) as the baseline and further excluded previous HCC cases before baseline. Of the 2878 participants that entered the cohort from 1989–1992, 1533 who were still alive and free of HCC continued to participate in follow-up examination after 1 January 2006, along with 767 participants who were also included in the main analysis (Supplementary Figure S1). The HCC occurrence of the 1533 subjects was followed up on until 31 December 2021.

## 3. Results

The baseline characteristics in participants with and without MAFLD are shown in Table 1. Of the 1453 eligible subjects (median age, 49.2 (IQR, 44.3–57.4) years; 4.6% diabetes; 31.3% obesity; 2.6% metabolic dysfunction), 775 (53.3%) had hepatic steatosis and 332 (22.9%) could be defined as MAFLD. In addition to more metabolic abnormalities, individuals with MAFLD were significantly more often cigarette smokers and had a history of excess alcohol consumption ≥ 140 g/week, both higher than those without MAFLD. Age, personal and family histories of liver disease, and variant distribution of the three fatty liver disease-related genes were not significantly different between individuals in the two groups. It is also worth noting that the prevalence of liver enzyme (ALT and AST) elevation was not different between the two groups.

### 3.1. HCC Risk

During the median follow-up of 19.3 years (IQR: 18.7–19.8), we identified 105 incident HCC cases with an annual incidence of 3.96 per 1000 person-years. There were a total of 219 deaths, including 82 individuals dying from liver-related disease and 137 individuals dying from extrahepatic causes of deaths (Table 1).

MAFLD was inversely associated with HCC after adjustment for age only (model 1) (Table 2). Because hepatic steatosis has been associated with a higher HBsAg seroclearance rate and a decreased risk of HCC in CHB [13,14], we further conducted the analysis to adjust for hepatic steatosis and any potential time-varying confounding by HBsAg seroclearance. The addition of the two variables markedly changed the HR associated with MAFLD from 0.63 (*p* = 0.0788) to 2.22 (*p* = 0.0454) (model 2), suggesting that the association between MAFLD and HCC risk was strongly influenced by hepatic steatosis and subsequent HBsAg seroclearance. To study the additional value of the MAFLD definition over its coincidental metabolic factors that are already taken into account in usual clinical practice, we also adjusted for the metabolic components of MAFLD, fatty liver disease-related genetic polymorphisms, and alcohol and tobacco use. As seen in model 3, the HR for MAFLD was reduced to 1.02 and was not statistically significant after adjustment for metabolic factors, whereas the association with HCC was stronger for diabetes (HR = 2.28, 95% CI = 1.10–4.74), obesity (HR = 1.72, 95% CI = 1.07–2.77), and metabolic dysfunction (HR = 3.30, 95% CI = 1.37–7.95) (all *p* < 0.03).

The study population composed mainly of untreated CHB patients, including only 98 (6.7%) participants who received nucleos(t)ide analogue or interferon treatment during follow-up (Table 1). MAFLD was not related to a higher HCC incidence after further adjustment for antiviral treatment. Besides metabolic abnormalities, the carrying of PNPLA3 (GG) or MBOAT7 (TT) homozygous variants was associated with increased HCC risk (Table 2). Further subgroup analyses showed that these fatty liver disease-associated variants did not affect the association of MAFLD and HCC (details are described in Supplementary Materials).

Additionally, we categorized MAFLD into three subtypes: MAFLD-diabetes (n = 44), MAFLD-obesity (n = 283), and MAFLD-metabolic dysfunction (nondiabetic, nonobese, and ≥ 2 other metabolic abnormalities, n = 5), and assessed whether the HCC risk differed between these subtypes. No MAFLD subtypes showed an increased risk of HCC (Supplementary Table S1).

### 3.2. Mediation Pathway Linking MAFLD and HCC

**Figure 2:**
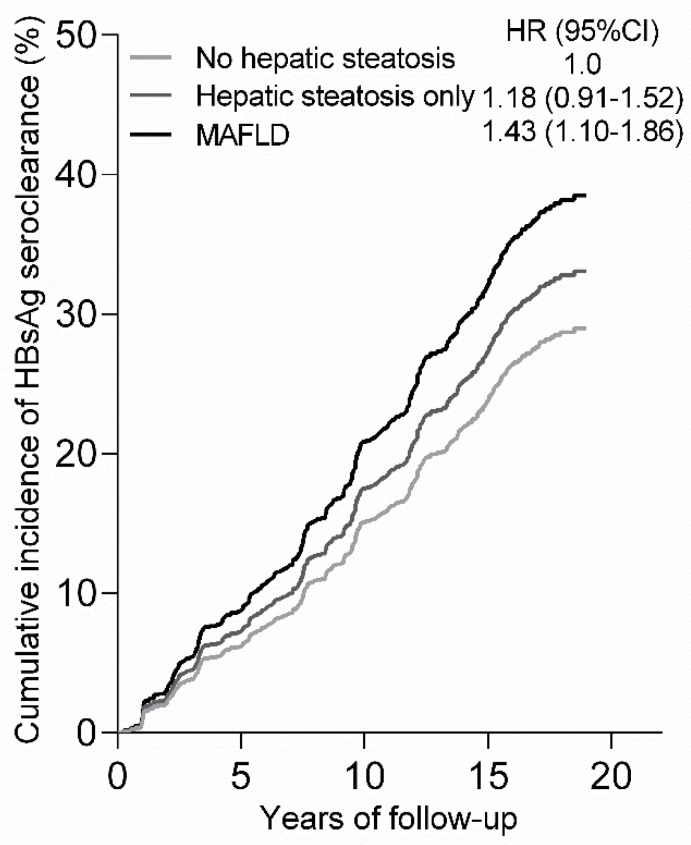
Cumulative incidence of HBsAg seroclearance. Cumulative incidence function was derived from a Cox’s model adjusted for age at recruitment.

During follow-up, HBsAg seroclearance was achieved in 336 subjects, and 95 and 241 incidences of HBsAg seroclearance, respectively, occurred in MAFLD and non-MAFLD participants (28.6% vs. 21.5%, *p* = 0.0069) (Table 1). The adjusted cumulative incidence function indicates that subjects with hepatic steatosis or MAFLD showed an increased probability of HBsAg seroclearance compared with those without hepatic steatosis, and MAFLD was more strongly associated with HBsAg seroclearance than hepatic steatosis only (Figure 2).

**Figure 3:**
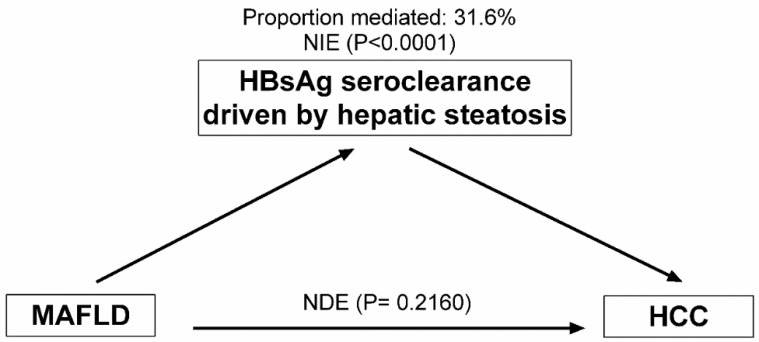
Directed acyclic graph of a structural model of mediation of the association between MAFLD and hepatocellular carcinoma (HCC) by HBsAg seroclearance driven by hepatic steatosis. The mediation analysis was performed using a bootstrapping procedure with 5000 resamples. NDE: natural direct effect; NIE: natural indirect effect.

Mediation analysis was then conducted to evaluate the extent to which the association between MAFLD and HCC was mediated via HBsAg seroclearance driven by hepatic steatosis at baseline. In mediation analysis, MAFLD was found to have no direct effect (*p* = 0.2160) on the risk of HCC. However, a significant indirect association (*p* for indirect effect <0.0001) was observed between MAFLD and HCC, indicating that 31.6% of the association of HCC with MAFLD was mediated by HBsAg seroclearance via hepatic steatosis at baseline (Figure 3).

### 3.3. Number of Metabolic Abnormality and HCC

**Figure 4:**
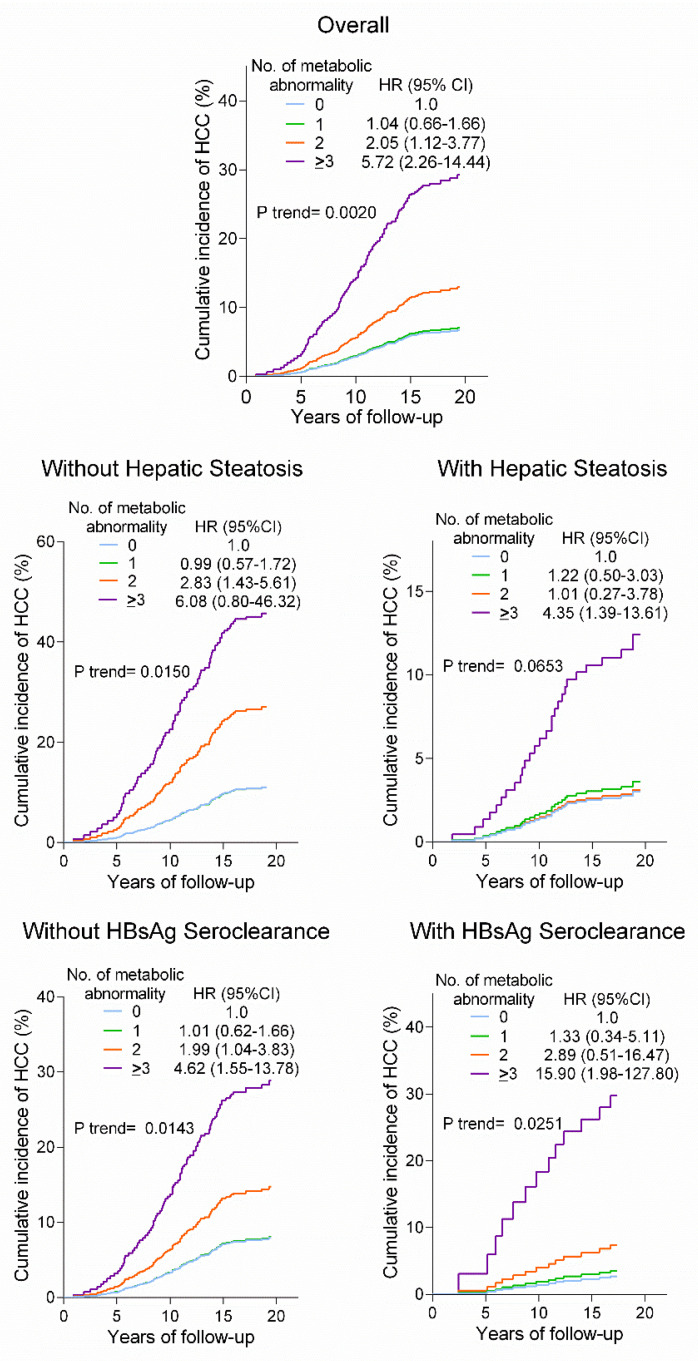
Absolute and relative risks of hepatocellular carcinoma (HCC) based on the number of metabolic abnormality (i.e., diabetes, obesity (BMI ≥ 25 kg/m^2^), high blood pressure, and hypertriglyceridemia). The cumulative incidence function and HRs (95% CIs) were derived from Cox models adjusted for age, hepatic steatosis, HBsAg seroclearance or not, genetic polymorphisms of PNPLA3, TM6SF2, and MBOAT7, and tobacco and alcohol use.

The adjusted cumulative incidences and HRs of HCC dramatically increased with an increasing number of metabolic abnormalities. Furthermore, a similarly high risk of HCC was observed in those with ≥3 risk abnormalities for subjects with and without hepatic steatosis, and for subjects with and without HBsAg seroclearance (Figure 4).

### 3.4. All-Cause and Extrahepatic Mortality

We also analyzed the associations of MAFLD with all-cause and extrahepatic mortality and found no increased risk associated with MAFLD (Supplementary Table S2).

### 3.5. Sensitivity Analysis

We performed a case-cohort analysis to examine the impacts of major viral factors on the MAFLD–HCC association in CHB. Supplementary Table S3 shows the baseline distributions of viral factors by MAFLD status in the case-cohort study. The analysis demonstrated a nonsignificant inverse association between MAFLD and HCC (HR = 0.51; *p* = 0.3048) after further adjustment for HBV genotype and viral load besides coincidental metabolic abnormalities and other covariates (model 3), but the link between coincidental metabolic abnormalities and increased risk for HCC remained significant and became even stronger (Supplementary Table S4).

Of the 1533 (baseline age: median (IQR) = 57.8 (52.9–64.8) years) subjects from the cohort 2006–2021, 444 (29.0%) had MAFLD at baseline, for which metabolic dysfunction was defined as ≥ 2 risk abnormalities, including hypertriglyceridemia, high blood pressure, and a waist circumference ≥ 90 cm. Over a median (IQR) follow-up of 13.3 (12.0–14.2) years, 64 HCC events occurred (Supplementary Table S5). The sensitivity analysis showed a similar association between MAFLD and HCC (model 3: adjusted HR = 0.73; *p* = 0.6538) (Supplementary Table S6) with the above findings.

## 4. Discussion

The results revealed that MAFLD was associated with a lower risk of HCC after adjustment for age only, and hepatic steatosis and subsequent development of HBsAg seroclearance played an important role as a mediating factor, accounting for 31.6% of the association of MAFLD and HCC. Although a twofold increased risk of HCC in MAFLD was observed after considering hepatic steatosis and HBsAg seroclearance in multivariable analyses, the association did not persist after additional adjustment for metabolic abnormalities. Instead, we found that after mutual adjustment for MAFLD and coincidental metabolic abnormalities, the associations between diabetes, obesity, and metabolic dysfunction and HCC remained, suggesting that the metabolic risk of MAFLD for HCC is mainly driven by metabolic abnormalities. Furthermore, the burden of metabolic risk abnormality was robustly associated with an increased risk of HCC, and this association was observed for participants both with and without hepatic steatosis and for participants both with and without HBsAg seroclearance.

Among patients with fatty liver disease who are mostly unaffected by viral hepatitis, it has been shown that MAFLD may be useful in identifying significant hepatic fibrosis [21,22]. However, data are limited on the associations between MAFLD and the natural history and advanced hepatic disease of CHB [8,23]. We found a significant association between MAFLD and a higher rate of HBsAg seroclearance, and the impact of MAFLD at baseline on the cumulative incidence of HBsAg loss was greater than that of hepatic steatosis alone, which has been previously reported to be associated with increased chance of HBsAg seroclearance [13,14]. MAFLD is a set of metabolic risk factors in which several metabolic components may be associated with HBsAg seroclearance [13]. Thus, the relatively greater impact of MAFLD vs. hepatic steatosis alone that was found might reflect the mixture effect of MAFLD. In addition to traditional multivariable regression modeling used for estimating the association of MAFLD on HCC risk, which does not allow for the evaluation of the causal chain, we sought to understand the pathway linking MAFLD, hepatic steatosis/HBsAg seroclearance, and consequent HCC using mediation analysis. The results indicated that hepatic steatosis and subsequent HBsAg loss, which is considered a functional cure of HBV infection and has been associated with a better prognosis of CHB [13,16,17], might account for a substantial proportion of the association of MAFLD with HCC, implicating the limitation of the use of the MAFLD definition for risk stratification for advanced stage liver disease (and even more so for HCC) during chronic HBV infection.

To the best of our knowledge, there are only two longitudinal/cohort studies that have investigated the clinical relevance of MAFLD for liver-related adverse outcomes in CHB, including a multiethnic liver-biopsy CHB cohort study and a longitudinal study based on serial liver stiffness measurements with data obtained from a tertiary Australian clinical center [8,23]. The liver-biopsy cohort study analyzed 1076 consecutive patients with CHB (mean age: 43.6 vs. 36.7 years for MAFLD vs. non-MAFLD), with a median follow-up of 9.8 years and 36 HCC incidents. MAFLD was found to be associated with a 1.93-fold increased risk of HCC after adjustment for age, sex, HBeAg status, advanced fibrosis, and antiviral treatment [8]. However, considering metabolic risk abnormalities included in the definition of MAFLD, such as diabetes and obesity (which have been associated with increased risk of HCC [11,12,20,24,25,26]), the effect of MAFLD observed in that study may be explained by the coincidental metabolic abnormalities. Otherwise, the study was limited by the small number of HCC events used and was based on more severe patients who underwent liver biopsies. Thus, further studies are required to re-examine the importance and impact of the MAFLD definition on the risk of HCC in CHB.

After adjustment for time-varying HBsAg seroclearance and metabolic risk factors, our findings of a lack of association between MAFLD and HCC are in line with a recent longitudinal analysis, which found no apparent worsening of hepatic fibrosis over time during a median follow-up period of 6 years in CHB patients with concurrent MAFLD, as compared with those without MAFLD [23]. In this analysis that used modelling to analyze serial liver stiffness measurements obtained from a total of 660 CHB patients (median age: 45.5 vs. 43.0 years for MAFLD vs. non-MAFLD) with adjustment for demographics, viral load, metabolic risk factors, and antiviral therapy, the majority of patients had detectable HBV DNA in serum and elevated ALT throughout follow-up, similar to the present work’s study population. Moreover, we extended the investigation of MAFLD and HCC to examine the impact of MAFLD subtypes based on diabetes, obesity, and metabolic dysfunction. Although the clinical manifestations and underlying pathophysiology can vary between these subtypes, our data did not suggest that any subtype of MAFLD could increase the risk of developing HCC.

Besides MAFLD and coincidental metabolic abnormalities, we also examined three major fatty liver disease-related genetic variants [18], which have been associated with steatosis/fibrosis/cirrhosis and HCC risk (mainly in Caucasians, especially the UK biobank cohort [27,28,29]), and found that the PNPLA3 rs738409 GG and MBOAT7 rs641738 TT homozygous missense variants were also associated with an increased risk of HCC in CHB. Due to the successful viral suppression that is expected to occur in antiviral drug-treated CHB patients, the impact of MAFLD on the risk of HCC might become more predominant. However, MAFLD was still not associated with a higher incidence of HCC after further adjustment for antiviral therapy or in the subgroups analyses stratified based on fatty liver disease-associated genetic background.

In contrast to MAFLD, which was not found to be associated with increased HCC risk, diabetes, obesity, and metabolic dysfunction were retained as main predictors of HCC after multivariable adjustment. Furthermore, the risk of HCC was strongly associated with the number of metabolic risk abnormalities present. The HR of HCC increased with each additional risk abnormality present from a nonsignificant 1.04-fold increase in the presence of one risk factor, to 5.72-fold in the presence of ≥ 3 risk factors. The gradually increasing risk of HCC with an increasing burden of metabolic abnormalities is consistent with the findings of our previous work using a similar cohort database [12], but the present results demonstrated higher hazards than those derived without taking hepatic steatosis and HBsAg seroclearance into account in the previous study. Compared with our previous work, an additional insight provided by the present work is that the cumulative effect of metabolic abnormalities presents a similarly high risk of HCC in subjects with and without hepatic steatosis, despite there being an inverse association between hepatic steatosis and HCC. More importantly, subjects both with and without HBsAg seroclearance were similarly susceptible to the aggregate effect of metabolic abnormalities, highlighting that assessment of metabolic abnormalities may help future therapies targeting the important factors that contribute most to decreasing the excess HCC incidence in CHB at different stages of its natural history.

The strengths of this study include the large sample size with a sufficient number of HCC events to provide robust relative and absolute estimates, the longitudinal natural history analysis of CHB, and ultrasound-diagnosed hepatic steatosis, resulting in higher accuracy in the diagnosis of MAFLD. The results were adjusted for possible important confounders, including fatty liver disease-related genetic predisposition and metabolic factors. Furthermore, we also assessed the robustness of the observed MAFLD–HCC association using a case-cohort analysis, which is an efficient design for a biomarker study with the retention of the temporal sequence of a prospective study [30], after taking account of the potential impacts of HBV genotype and viral load.

However, several limitations have to be taken into consideration. First, we used baseline measurements. The prevalence of metabolic disorder increases with increasing age. The HR estimates for MAFLD/metabolic factors may therefore depend on the initial age of the subjects and the length of the total time period under consideration, since more subjects may be misclassified when the follow-up time is long, which could cause an underestimation. However, we also analyzed the 2006–2021 longitudinal cohort dataset (n = 1533), which showed similar results. Second, because our study used data already collected, the definition of MAFLD was modified to accommodate the data. In this study, not all metabolic risk factors were collected at baseline; however, our sensitivity analysis using a stricter MAFLD definition based on the data with waist circumference as part of the follow-up examination also showed that MAFLD was not associated with a higher incidence of HCC. Third, because all the study subjects were male, our results should be interpreted with caution when applied to females. However, because HCC is two to three times more frequent in men than in women, a larger cohort of CHB patients and a longer follow-up time are needed for a similar study in females. Finally, we did not obtain information pertaining to drug use for the treatment of dysmetabolic traits (such as statins or metformin), which has been associated with a reduced risk of HCC, although limited data exist on HCC in the context of NAFLD specifically [31,32].

## 5. Conclusions

In conclusion, the results of the present work show that there is no increased risk of HCC for MAFLD, defined as hepatic steatosis in the presence of metabolic abnormality, in CHB after a substantial 19 years of follow-up. Additionally, MAFLD was not found to be associated with increased risk for either all-cause or extrahepatic mortality in CHB, as previously reported in uninfected people [4,5]. During chronic HBV infection, the association between MAFLD and HCC appears to be largely mediated by the development of HBsAg seroclearance driven by hepatic steatosis. An increased HCC risk was; however, seen for metabolic abnormalities. The number of metabolic risk factors provides a more informative graded assessment of risk for HCC in CHB, regardless of hepatic steatosis or HBsAg seroclearance.

## Figures and Tables

**Table 1:** Baseline characteristics of subjects by MAFLD status.

	MAFLD (n = 332)		No MAFLD (n = 1121)		
Variable	N	(%)	n	(%)	*p* ^a^
Age (years), median (IQR)	49.5	(44.3–57.0)	49.0	(44.3–57.5)	0.6405
Cigarette smoking					0.0011
Never	199	(59.9)	780	(69.6)	
Past	56	(16.9)	117	(10.4)	
Current	77	(23.2)	224	(20.0)	
Alcohol drinking	94	(28.3)	228	(20.3)	0.0021
Alcohol intake ≥ 140 g/week ^b^	41	(12.5)	81	(7.3)	0.0030
History of liver disease	19	(5.7)	77	(6.9)	0.4603
First-degree family history of HCC ^b^	19	(5.7)	76	(6.8)	0.4916
Initial ultrasonography					
Hepatic steatosis	332	(100)	443	(39.5)	<0.0001
Liver cirrhosis	3	(0.9)	78	(7.0)	<0.0001
Metabolic risk factors					
BMI (kg/m^2^), median (IQR)	26.3	(25.4–27.7)	23.1	(21.7–24.3)	<0.0001
Obesity (BMI ≥ 25 kg/m^2^) ^c^	302	(91.0)	153	(13.7)	<0.0001
Impaired fasting glucose	102	(30.7)	254	(22.7)	0.0027
Diabetes	44	(13.3)	23	(2.1)	<0.0001
Hypertriglyceridemia	97	(29.2)	106	(9.5)	<0.0001
HyperHypercholesterolemia	22	(6.6)	83	(7.4)	0.6308
High blood pressure	70	(21.1)	113	(10.1)	<0.0001
Liver enzyme ^b^					
ALT ≥ 35 U/L	30	(9.0)	104	(9.3)	0.8867
AST ≥ 35 U/L	25	(7.5)	76	(6.8)	0.6453
Anti-HCV	16	(4.9)	66	(6.1)	0.5004
PNPLA3 rs738409 ^b^					0.7590
CC	125	(37.7)	434	(38.8)	
CG	162	(48.8)	522	(46.6)	
GG	45	(13.6)	164	(14.6)	
TM6SF2 rs58542926 ^b^					0.2105
CC	281	(84.6)	974	(87.0)	
CT	50	(15.1)	135	(12.1)	
TT	1	(0.3)	11	(1.0)	
MBOAT7 rs641738 ^b^					0.6639
CC	181	(54.5)	637	(56.9)	
CT	128	(38.6)	417	(37.2)	
TT	23	(6.9)	66	(5.9)	
Follow-up					
HBsAg seroclearance	95	(28.6)	241	(21.5)	0.0069
Antiviral therapy	18	(5.4)	80	(7.1)	0.3194
HCC events	17	(5.1)	88	(7.9)	0.0916
All-cause deaths	45	(13.6)	174	(15.5)	0.3788
Liver-related	14	(4.2)	68	(6.1)	0.2246
Extrahepatic	31	(9.3)	106	(9.5)	0.9483

ALT: alanine aminotransferase; anti-HCV: hepatitis C virus antibody; AST: aspartate aminotransferase; HCC: hepatocellular carcinoma; IQR: interquartile range. ^a^ Derived from χ^2^ or Fisher’s exact test for categorical variables, and Wilcoxon rank-sum test for continuous variables. ^b^ Data not available for all participants. Missing information in participants with MAFLD: amount of alcohol consumed (n = 3) and anti-HCV (n = 6); missing information in participants without MAFLD: amount of alcohol consumed (n = 8), family history of HCC (n = 1), ALT (n = 2), AST (n = 3), anti-HCV (n = 42) and genetic polymorphisms (n = 1). ^c^ Defined according to the Asian criteria.

**Table 2:** Adjusted hazard ratios of risk factors for incident hepatocellular carcinoma.

		Model 1 ^a^		Model 2 ^b^		Model 3 ^c^	
Variable	HCC, n	HR (95% CI)	*p*	HR (95% CI)	*p*	HR (95% CI)	*p*
MAFLD							
No (n = 1121)	88	1.0		1.0		1.0	
Yes (n = 332)	17	0.63 (0.37–1.06)	0.0788	2.22 (1.02–4.86)	0.0454	1.02 (0.40–2.59)	0.9698
Hepatic steatosis							
No (n = 678)	78	1.0		1.0		1.0	
Yes (n = 775)	27	0.30 (0.19–0.46)	<0.0001	0.20 (0.10–0.39)	<0.0001	0.23 (0.12–0.46)	<0.0001
HBsAg seroclearance ^d^							
No (n = 1117)	92	1.0		1.0		1.0	
Yes (n = 336)	13	0.47 (0.24–0.94)	0.0319	0.50 (0.25–0.99)	0.0470	0.50 (0.25–0.99)	0.0483
Diabetes							
No (n = 1386)	96	1.0				1.0	
Yes (n = 67)	9	1.71 (0.86–3.41)	0.1260			2.28 (1.10–4.74)	0.0268
Obesity							
No (n = 998)	67	1.0				1.0	
Yes (n = 455)	38	1.22 (0.82–1.81)	0.3378			1.72 (1.07–2.77)	0.0251
Metabolic dysfunction							
No (n = 1415)	99	1.0				1.0	
Yes (n = 38)	6	2.32 (1.02–5.30)	0.0461			3.30 (1.37–7.95)	0.0077
PNPLA3							
CC (n = 559)	33	1.0				1.0	
CG (n = 684)	50	1.25 (0.80–1.93)	0.3278			1.40 (0.90–2.19)	0.1378
GG (n = 209)	22	1.91 (1.12–3.28)	0.0185			2.59 (1.49–4.49)	0.0007
TM6SF2							
CC (n = 1255)	92	1.0				1.0	
CT + TT (n = 197)	13	0.87 (0.49–1.56)	0.6435			1.03 (0.57–1.85)	0.9174
MBOAT7							
CC (n = 818)	52	1.0				1.0	
CT (n = 545)	43	1.28 (0.86–1.92)	0.2307			1.28 (0.85–1.93)	0.2311
TT (n = 89)	10	1.88 (0.95–3.70)	0.0683			1.89 (0.95–3.74)	0.0691

HCC: hepatocellular carcinoma. HR: hazard ratio. CI: confidence interval. Note. One subject had missing data on genetic polymorphisms. ^a^ Adjusted for age at recruitment. ^b^ Adjusted for age at recruitment and variables listed in the table. ^c^ Adjusted for age at recruitment, tobacco and alcohol use, and variables listed in the table. ^d^ HBsAg seroclearance was treated as a time-varying covariate in the model.

## Data Availability

The datasets generated and/or analyzed during the current study are not publicly available in accordance with the policy of the Health and Welfare Data Science Center, Ministry of Health and Welfare, Taiwan, but are available from the corresponding author upon reasonable request.

## Supplementary Materials

The following supporting information can be downloaded at: https://www.mdpi.com/article/10.3390/cancers14236012/s1, Figure S1: Derivation of the main cohort and the cohort dataset used for the sensitivity analysis. 767 subjects overlapped between the two cohort datasets.; Table S1: Association between subtypes of MAFLD and incident hepatocellular carcinoma; Table S2: MAFLD and hazard ratios of all-cause and extrahepatic mortality; Table S3: Distribution of major viral factors by MAFLD status at baseline: Case-cohort analysis (HCC: 71; controls: 522); Table S4: analysis of HCC risk associated with MAFLD after adjustment for HBV genotype and viral load: Case-cohort analysis; Table S5: Baseline characteristics of subjects with and without MAFLD (Database 2006–2021, n = 1533); Table S6: Adjusted hazard ratios of risk factors for incident hepatocellular carcinoma (Database 2006–2021, n = 1533). References [18,20] are cited in the supplementary material.

## Abbreviations

ALT, alanine aminotransferase; anti-HBs, hepatitis B surface antibody; anti-HCV, hepatitis C virus antibody; AST, aspartate aminotransferase; BMI, body mass index; CHB, chronic hepatitis B; CI, confidence interval; HBsAg, hepatitis B surface antigen; HBV, hepatitis B virus; HCC, hepatocellular carcinoma; HR, hazard ratio; IQR, interquartile range; MAFLD, metabolic-associated fatty liver disease; NHIRD, National Health Insurance Research Database; NAFLD, nonalcoholic fatty liver disease.
