# Supplementary material for: Metabolic-Associated Fatty Liver Disease, Hepatitis B Surface Antigen Seroclearance, and Long-Term Risk of Hepatocellular Carcinoma in Chronic Hepatitis B
Source: Cancers (Basel). 2022 Dec 6;14(23):6012. doi: 10.3390/cancers14236012 (PMC9736898; doi:10.3390/cancers14236012)
Supplement: Supplementary file 1 [file cancers-14-06012-s001.zip › Supplementary Figure S1.pdf]

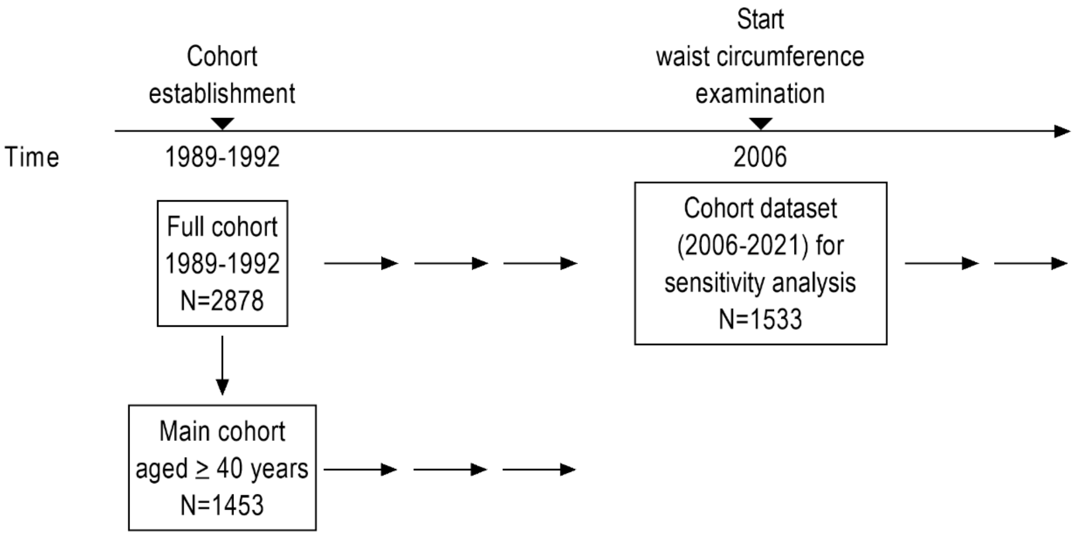

Figure S1. Derivation of the main cohort and the cohort dataset used for the sensitivity analysis. 767 subjects overlapped between the two cohort datasets.
