# Supplementary material for: Metabolic-Associated Fatty Liver Disease, Hepatitis B Surface Antigen Seroclearance, and Long-Term Risk of Hepatocellular Carcinoma in Chronic Hepatitis B
Source: Cancers (Basel). 2022 Dec 6;14(23):6012. doi: 10.3390/cancers14236012 (PMC9736898; doi:10.3390/cancers14236012)
Supplement: Supplementary file 1 [file cancers-14-06012-s001.zip › Supplementary Materials MAFLD MS 1015 2022.pdf]

Metabolic-Associated Fatty Liver Disease, Hepatitis B Surface Antigen Seroclearance, and Long-Term Risk of Hepatocellular Carcinoma in Chronic Hepatitis B

Ming-Whei Yu, Chih-Lin Lin, Chun-Jen Liu, Wan-Jung Wu, Jui-Ting Hu, Yi-Wen Huang

Supplementary Results

After further adjustment for antiviral treatment besides covariates in model 3 listed in Table 2, the HR of HCC associated with MAFLD was estimated to be 1.17 (95% CI: 0.47-2.96; p = 0.7354).

We also examined whether the association between MAFLD and HCC is influenced by genetic susceptibility to fatty liver disease. Effect modification was tested by fitting an interaction term between MAFLD and the genetic polymorphism studied. In interaction analyses, PNPLA3 rs738409 C/G, TM6SF2 rs58542926 C/T, and MBOAT7 rs641738 C/T genotypes were coded 0, 1, and 2 according to the number of fatty liver disease-associated risk allele based on previous studies.[18] After adjustment for covariates in model 3 listed in Table 2, the results showed no notable interaction between MAFLD and PNPLA3 (p for interaction = 0.7398), TM6SF2 (p for interaction = 0.2333), or MBOAT7 (p for interaction = 0.3440).

We further assessed the joint association between MAFLD and genetic susceptibility by deriving a combined variable with 4 groups: non-MAFLD/low genetic susceptibility (0 risk allele), MAFLD/low genetic susceptibility, non-MAFLD/high genetic susceptibility (≥1 risk allele), and MAFLD-high genetic susceptibility. Across all joint analyses, the combination of MAFLD and high genetic susceptibility was not observed to increase HCC risk. For example, the HRs of HCC for the combinations of MAFLD and PNPLA3 were 1.50 (95% CI = 0.48–4.71, p = 0.4892), 1.80 (95% CI = 1.13-2.86, p = 0.0133), and 1.49 (95% CI = 0.52–4.25, p = 0.4544), respectively, for MAFLD/low genetic susceptibility, non-MAFLD/high genetic susceptibility, and MAFLD-high genetic susceptibility, as compared with non-MAFLD/low genetic susceptibility.

Case-cohort study

We used data from a previous case-cohort study, involving a total of 1143 CHB patients nested in the GECC cohort, to assess the impacts of HBV genotype and viral load on the estimated effect of MAFLD on the risk of HCC.[20] In the present study, we included all of the HCC cases (n = 71; 33 outside the subcohort) and controls (n = 522) aged ≥40 years who were included in the cohort analysis of MAFLD and HCC.

Supplementary Table 3 shows the distributions of major viral factors at baseline in individuals with vs. without MAFLD. As can be seen, HBeAg, viral load, and HBV genotype did not significantly differ between individuals with and without MAFLD, although MAFLD patients tended to have a lower prevalence of genotype C HBV infection than patients without MAFLD (12.0% vs. 18.6%, p = 0.0751). We then conducted weighted Cox regression to calculate HRs (95% CIs) of HCC for MAFLD and coincidental metabolic abnormalities after adjustment for HBV genotype and viral load. The results still showed no increased risk of HCC for MAFLD (HR = 0.51; 95% CI = 0.14–1.86) after further adjustment for the two major viral factors (HBV genotype C vs. non-C: HR= 4.42 [2.47–7.88]; HBV-DNA log copies/mL: HR= 1.32 [1.18–1.49]) besides coincidental metabolic abnormalities and other covariates (model 3). However, the associations with HCC for coincidental metabolic abnormalities, including diabetes (HR = 3.95, 95% CI = 1.37-11.38), obesity (HR = 3.03, 95% CI = 1.56-

5.87), and metabolic dysfunction (HR = 4.64, 95% CI = 1.72–12.50), became even stronger (Supplementary Table 4).

Table S1. Association between subtypes of MAFLD and incident hepatocellular carcinoma.

Variable	HCC, n	Model 1[a] HR (95% CI)	p	Model 2[b] HR (95% CI)	p	Model 3[c] HR (95% CI)	p
No MAFLD (n = 1121)	88	1.0		1.0		1.0	
MAFLD-obesity or metabolic dysfunction (n = 288)	13	0.57 (0.32–1.01)	0.0545	1.99 (0.87–4.54)	0.1015	0.96 (0.36–2.55)	0.9388
MAFLD-diabetes (n = 44)	4	0.99 (0.36–2.69)	0.9763	3.58 (1.12–11.47)	0.0316	1.31 (0.29–5.98)	0.7239

Note: No HCC events occurred in 5 MAFLD-metabolic dysfunction only (non-obese, non-diabetic, ≥2 other risk abnormalities). HCC: hepatocellular carcinoma, HR: hazard ratio, CI: confidence interval. [a]Adjusted for age at recruitment. [b]Adjusted for age at recruitment, hepatic steatosis, and time-varying HBsAg seroclearance. [c]Adjusted for age at recruitment, hepatic steatosis, time-varying HBsAg seroclearance, tobacco and alcohol use, diabetes, obesity, metabolic dysfunction, and genetic polymorphisms of PNPLA3, TM6SF2, and MBOAT7.

Table S2. MAFLD and hazard ratios of all-cause and extrahepatic mortality.

Variable	Events, n	Model 1[a] HR (95% CI)	Model 2[b] HR (95% CI)	Model 3[c] HR (95% CI)
All-cause mortality				
No MAFLD	174	1.0	1.0	1.0
MAFLD	45	0.84 (0.60–1.16)	1.41 (0.92–2.18)	0.81 (0.45–1.43)
Extrahepatic mortality[d]				
No MAFLD	106	1.0	1.0	1.0
MAFLD	31	0.96 (0.64–1.43)	1.07 (0.66–1.74)	0.73 (0.34–1.55)

HR: hazard ratio. CI: confidence interval. [a]Adjusted for age at recruitment. [b]Adjusted for age at recruitment, hepatic steatosis, and time-varying HBsAg seroclearance. [c]Adjusted for age at recruitment, hepatic steatosis, time-varying HBsAg seroclearance, tobacco and alcohol use, diabetes, obesity, metabolic dysfunction, and genetic polymorphisms of PNPLA3, TM6SF2, and MBOAT7. [d]HRs and 95% CIs were derived from competitive risk Cox regression models, with liver-related deaths considered as a competing event.

Table S3. Distribution of major viral factors by MAFLD status at baseline: Case-cohort analysis (HCC: 71; controls: 522).

Variable	MAFLD (n = 134) n	(%)	No MAFLD (n = 459) n	(%)	p[a]
Age (years), median (IQR)	49.4	(44.7–55.0)	48.9	(44.1–57.6)	0.8397
HBeAg positive[b]	7	(5.3)	29	(6.5)	0.6035
HBV DNA log copies/mL, median (IQR)	4.14	(3.04–5.39)	3.99	(3.10–5.56)	0.7722
HBV DNA≥ 4 log copies/mL	71	(53.0)	229	(49.9)	0.5285
HBV genotype C[b]	16	(12.0)	85	(18.6)	0.0751
Liver enzyme[b]					
ALT ≥ 35 U/L	11	(8.2)	38	(8.3)	0.9687
AST ≥ 35 U/L	8	(6.0)	30	(6.6)	0.8007

Initial ultrasonography					
Hepatic steatosis	134	(100)	163	(35.5)	<0.0001
Metabolic risk factors					
Obesity BMI≥ 25 kg/m^2)[c]	120	(89.6)	66	(14.4)	<0.0001
Diabetes	19	(14.2)	10	(2.2)	<0.0001
Hypertriglyceridemia	38	(28.4)	47	(10.2)	<0.0001
Hypercholesterolemia	5	(3.7)	34	(7.4)	0.1657
High blood pressure	32	(23.9)	45	(9.8)	<0.0001
**Follow-up**					
HBsAg seroclearance	37	(27.6)	95	(20.7)	0.0905
HCC events	12	(9.0)	59	(12.9)	0.2213

ALT: alanine aminotransferase; AST: aspartate aminotransferase; HCC: hepatocellular carcinoma; IQR: interquartile range. [a]Derived from χ^2 or Fisher's exact test for categorical variables, and Wilcoxon rank-sum test for continuous variables. [b]Data not available for all participants. Missing information in participants with MAFLD: HBeAg (n = 1) and HBV genotype (n = 1); missing information in participants without MAFLD: HBeAg (n = 13), HBV genotype (n = 3), ALT (n = 2), and AST (n = 3). [c]Defined according to the Asian criteria.

Table S4. Weighted Cox regression analysis of HCC risk associated with MAFLD after adjustment for HBV genotype and viral load: Case-cohort analysis.

	**Model 1[a]**		Model 2[b]		Model 3[c]	
**Variable**	**HR (95% CI)**	**p**	HR (95% CI)	p	HR (95% CI)	p
MAFLD	0.68 (0.36–1.27)	0.2236	2.22 (0.83–5.96)	0.1118	0.51 (0.14–1.86)	0.3048
Hepatic steatosis	0.37 (0.21–0.63)	0.0003	0.31 (0.13–0.72)	0.0062	0.39 (0.16–0.98)	0.0442
HBsAg seroclearance	0.44 (0.22–0.89)	0.0231	0.49 (0.22–1.07)	0.0747	0.53 (0.23–1.19)	0.1242
Diabetes	1.70 (0.73–3.95)	0.2214			3.95 (1.37–11.38)	0.0109
Obesity	1.73 (1.06–2.81)	0.0284			3.03 (1.56–5.87)	0.0011
Metabolic dysfunction	3.01 (1.16–7.83)	0.0239			4.64 (1.72–12.50)	0.0024
HBV DNA log copies/mL (continuous)	1.40 (1.25–1.56)	<0.0001	1.26 (1.12–1.42)	<0.0001	1.32 (1.18–1.49)	<0.0001
HBV genotype C (vs. Non-C)	5.04 (2.97–8.55)	<0.0001	4.57 (2.67–7.83)	<0.0001	4.42 (2.47–7.88)	<0.0001

HCC: hepatocellular carcinoma. HR: hazard ratio. CI: confidence interval. [a]Adjusted for age at recruitment. [b]Adjusted for age at recruitment and variables listed in the table. [c]Adjusted for age at recruitment, tobacco and alcohol use, genetic polymorphisms of PNPLA3, TM6SF2, and MBOAT7, and variables listed in the table.

Table S5. Baseline characteristics of subjects with and without MAFLD (Database 2006–2021, n = 1533).

	**MAFLD (n = 444)**		**No MAFLD (n = 1089)**		
**Variable**	**n**	**(%)**	**n**	**(%)**	**p[a]**
Age (years), median (IQR)	57.0	(52.5–63.1)	58.2	(53.0–65.4)	0.0087
Cigarette smoking					0.0595
Never	304	(68.5)	808	(74.3)	
Past	40	(9.0)	73	(6.7)	
Current	100	(22.5)	207	(19.0)	
Alcohol drinking	105	(23.7)	187	(17.2)	0.0034
Alcohol intake ≥140 g/week	40	(9.1)	63	(5.8)	0.0207
Ultrasonography					
Hepatic steatosis	444	(100)	184	(16.9)	<0.0001
Liver cirrhosis	3	(0.7)	33	(3.0)	0.0045
Metabolic risk factors					
BMI (kg/m^2), median (IQR)	26.5	(25.4–28.0)	23.3	(21.9–24.7)	<0.0001
Obesity (BMI≥ 25 kg/m^2)[b]	373	(84.0)	208	(19.1)	<0.0001

Central obesity (waist circumference≥90 cm)	254	(57.2)	148	(13.6)	<0.0001
Impaired fasting glucose	93	(21.0)	182	(16.7)	0.0500
Diabetes	116	(26.1)	94	(8.6)	<0.0001
Hypertriglyceridemia	130	(29.3)	104	(9.6)	<0.0001
High blood pressure	210	(47.3)	241	(22.1)	<0.0001
Metabolic dysfunction (≥2 risk abnormalities, including hypertriglyceridemia, high blood pressure, and waist circumference ≥90 cm[b])	197	(44.4)	71	(6.5)	<0.0001
ALT ≥ 35 U/L	129	(29.1)	222	(20.4)	0.0002
PNPLA3 rs738409					0.1316
CC	159	(35.9)	429	(39.5)	
CG	208	(47.0)	512	(47.1)	
GG	76	(17.2)	146	(13.4)	
TM6SF2 rs58542926					0.6634
CC	384	(86.7)	933	(85.8)	
CT+TT	59	(13.3)	154	(14.2)	
MBOAT7 rs641738					0.1209
CC	234	(52.8)	614	(56.5)	
CT	185	(41.8)	397	(36.5)	
TT	24	(5.4)	76	(7.0)	
**Follow-up**					
HBsAg seroclearance	200	(45.1)	351	(32.2)	<0.0001
HCC events	12	(2.7)	52	(4.8)	0.0682
All-cause deaths	38	(8.6)	98	(9.0)	0.7832
Liver-related	5	(1.1)	15	(1.4)	0.8081
Extrahepatic	33	(7.4)	83	(7.6)	0.8989

Note: Data not available for all participants. Missing information in participants with MAFLD: amount of alcohol consumed (n = 5) and genetic polymorphisms (n = 1); missing information in participants without MAFLD: cigarette smoking (n = 1), amount of alcohol consumed (n = 7), and genetic polymorphisms (n = 2). ALT: alanine aminotransferase; HCC: hepatocellular carcinoma; IQR: interquartile range. [a]Derived from χ^2 or Fisher's exact test for categorical variables, and Wilcoxon rank-sum test for continuous variables. [b]Defined according to the Asian criteria.

Table S6. Adjusted hazard ratios of risk factors for incident hepatocellular carcinoma (Database 2006–2021, n = 1533).

		Model 1[a]		Model 2[b]		Model 3[c]	
**Variable**	**HCC, n**	**HR (95% CI)**	**p**	**HR (95% CI)**	**P**	**HR (95% CI)**	**p**
MAFLD							
No (n = 1089)	52	1.0		1.0		1.0	
Yes (n = 444)	12	0.59 (0.31–1.10)	0.0963	1.68 (0.47–5.96)	0.4213	0.73 (0.18–2.94)	0.6538
Hepatic steatosis							
No (n = 905)	49	1.0		1.0		1.0	
Yes (n = 628)	15	0.46 (0.25–0.81)	0.0080	0.31 (0.10–1.01)	0.0517	0.40 (0.12–1.34)	0.1368
HBsAg seroclearance [d]							
No (n = 982)	53	1.0		1.0		1.0	
Yes (n = 551)	11	0.19 (0.03–1.37)	0.0998	0.20 (0.03–1.45)	0.1110	0.18 (0.03–1.33)	0.0936
Diabetes							
No (n = 1323)	51	1.0				1.0	
Yes (n = 210)	13	1.55 (0.84–2.86)	0.1660			1.98 (1.03–3.78)	0.0395
Obesity (BMI≥ 25 kg/m^2)							
No (n = 952)	37	1.0				1.0	
Yes (n = 581)	27	1.33 (0.81–2.20)	0.2627			2.03 (1.12–3.69)	0.0194

Metabolic dysfunction[e]							
No (n = 1265)	53	1.0				1.0	
Yes (n = 268)	11	0.99 (0.51–-1.89)	0.9627			1.05 (0.50–2.19)	0.9047
PNPLA3							
CC (n = 588)	17	1.0				1.0	
CG (n = 720)	37	1.83 (1.03–3.24)	0.0401			1.87 (1.05–3.33)	0.0333
GG (n = 222)	10	1.56 (0.71–3.41)	0.2649			1.92 (0.87–4.22)	0.1054
TM6SF2							
CC (n = 1317)	55	1.0				1.0	
CT+TT (n = 213)	9	0.98 (0.48–1.98)	0.9485			1.08 (0.53–2.20)	0.8410
MBOAT7							
CC (n = 848)	39	1.0				1.0	
CT (n = 582)	21	0.78 (0.46–1.33)	0.3656			0.83 (0.48–1.41)	0.4796
TT (n = 100)	4	0.88 (0.31–2.46)	0.8044			0.93 (0.33–2.64)	0.8946

HCC: hepatocellular carcinoma. HR: hazard ratio. CI: confidence interval. Note. 3 subjects with missing data on genetic polymorphisms. [a]Adjusted for age at recruitment. [b]Adjusted for age at recruitment and variables listed in the table. [c]Adjusted for age at recruitment, tobacco and alcohol use, and variables listed in the table. [d]HBsAg seroclearance was treated as a time-varying covariate in the model. [e] ≥2 risk abnormalities, including hypertriglyceridemia, high blood pressure, and waist circumference ≥90 cm.
